# Can Antibiotic Stewardship Be Achieved by Utilizing the Stewardship Through Educating Patients (S.T.E.P.) Process?

**DOI:** 10.7759/cureus.47992

**Published:** 2023-10-30

**Authors:** Kathy L Havlicek, Ahmed Muhsin

**Affiliations:** 1 Family Medicine, Oceania University of Medicine, Apia, WSM; 2 Research, Oceania University of Medicine, Apia, WSM

**Keywords:** appropriate antibiotic prescribing, antibiotic stewardship through educating patients, interventional study, quality improvement research, heathcare education, antibiotic resistance programs, antibiotic resistance, antibiotic prescribing, antibiotic stewardship education, research projects in antibiotic stewardship programme

## Abstract

Background: Despite research and efforts to mitigate bacterial resistance, antibiotic overprescribing continues to occur, often due to real or perceived expectations of patients.

Objective: The purpose of this study was to determine: (1) if there's an association between the provider's patient education efforts and the patient's satisfaction, and (2) the research participant's subsequent behavior concerning antibiotic prescriptions, by utilizing the Stewardship Through Educating Patients (S.T.E.P.) process for positive prescriptive change. The S.T.E.P. program features straightforward, simple education via face-to-face counseling at patient encounters, along with presenting applicable printed educational pamphlets from the Centers for Disease Control and Prevention (CDC). These two interventions were utilized in this study with research participants from the healthcare provider, with education focused on appropriate antibiotic use in the treatment of adults diagnosed with common illnesses such as an upper respiratory infection (the common cold), acute sinusitis, and acute bronchitis, which oftentimes are viral in origin.

Method: This Quality Improvement (QI) interventional study utilized the researcher's direct face-to-face patient education and CDC printed materials as a measure of antibiotic prescribing as a primary outcome, with patient satisfaction as a secondary outcome via convenience sampling of 40 hospital employees who utilized a free hospital-based employee healthcare clinic.

Results: Patient-teaching by this study's researcher, along with supplemental printed patient education material from the CDC that were given to research participants during an initial medical encounter, were effective interventions used in reducing antibiotic prescribing, as evidenced by a positive patient satisfaction in 95% of research participants.

Conclusions: Antibiotic use in the treatment of adults diagnosed with common illnesses such as an upper respiratory infection (the common cold), acute sinusitis, and acute bronchitis, may be safely reduced by using a combination of patient-education and clinician intervention.

## Introduction

Despite efforts to curb the overuse of antibiotics, especially in light of the growing antibiotic resistance concerns, physicians and other health care providers continue to prescribe antibiotics at high frequencies, and sometimes quite inappropriately because of real or perceived bias from patients and others. While there is pressure to not prescribe antibiotics, the health care provider is also under pressure by their patients to prescribe them. Time constraints also factor in, as sometimes it’s easier (and faster) for physicians to just give the patient what they want (a perceived antibiotic prescription) and move on to the next patient or task at hand. Many past and current educational programs have been aimed at changing prescriber habits through various antibiotic stewardship programs. Perhaps it’s time to focus on the patient instead of prescribers, through educational efforts as a way to protect and preserve our currently available antibiotics from overuse and the ongoing threat of antibiotic resistance. This scholarly activity will focus on the clinical controversy involving antibiotic over-prescribing and inappropriate prescribing, based on best practice guidelines for antibiotic use, and the effectiveness of direct patient influence through the Stewardship Through Educating Patients (S.T.E.P.) process for positive prescriptive change in an out-patient clinical setting.

Over the years antibiotic resistance has become a global health threat worldwide. High levels of antibiotic resistance are now commonly seen throughout the world [1-6]. Of heightened concern is that new resistance mechanisms are also emerging and spreading globally, which threatens our ability to treat infectious diseases, including common illnesses [1-3]. Antibiotic resistance is accelerated by the misuse and overuse of antibiotics, as well as poor infection prevention and control, and the ability to purchase antibiotics as over-the-counter (OTC) medications in some countries around the world [1,7,8]. Healthcare providers can play a key role in antibiotic stewardship efforts to implement best practices in antimicrobial use, however, to date, the results of changing prescriber habits have often been slow and non-sustaining, despite efforts put forth by reputable forces of change, including the World Health Organization (WHO) and the Centers for Disease Control and Prevention (CDC) in the United States.

This research project utilized the S.T.E.P. process, with brief, yet effective educational efforts delivered at the direct patient care level in an outpatient primary care clinic setting, rather than focusing on the prescriber, as a means of enhancing antibiotic stewardship via curbing antibiotic overuse and resistance. The S.T.E.P. program features straightforward, simple education via face-to-face counseling at patient encounters, along with presenting applicable printed educational pamphlets from the CDC. These two interventions were utilized in this study with research participants from the health care provider, with education focused on appropriate antibiotic use in the treatment of adults diagnosed with common illnesses such as an upper respiratory infection (the common cold), acute sinusitis, and acute bronchitis, which oftentimes are viral in origin.

Healthcare professionals generally deal directly with patients and therefore can have access and influence over patient-teaching content and discussion. Physicians, nurse practitioners, and physician assistants who have prescribing authority can serve as educators to affect change in their patients, and are often seen as knowledgeable experts when it comes to designing and implementing treatment interventions, specific to each patient. The prescriber can also work with their patients to help prevent and control the spread of antibiotic resistance by focusing on positive and preventative healthcare measures. This can be realized but it generally necessitates a shifting paradigm from treating disease to preventing disease and health promotion. This is not only a desirable focus, but one that is necessary for cost-containment, as well as antibiotic stewardship by decreasing the need for antibiotic therapy.

To prevent the overuse of antibiotics and hence control the spread of antibiotic resistance, prescribers can encourage patients to: (1) focus on prevention of illness via good handwashing, (2) cover one’s nose and mouth when coughing or sneezing, (3) promote healthy lifestyles, including diet, exercise, adequate sleep, and limiting stress, (4) utilize and keep their immunization status current, including obtaining annual influenza vaccines, (5) maintain a clean, safe, healthy living environment.

Healthcare providers can (1) be cognizant to only prescribe antibiotics when they are absolutely necessary, according to current best-practice prescribing guidelines, (2) talk to patients about appropriate antibiotic use, antibiotic resistance, and the health concerns/side effects of antibiotic over-use and misuse, (3) monitor local antibiotic resistance rates and report antibiotic-resistant infections to appropriate and authorized surveillance members of the local community (local health departments, etc.) and broader authorities, as applicable, (4) utilize patient-teaching to educate patients about appropriate antibiotic use and antibiotic stewardship, especially in common illnesses that tend to be viral in nature, such as acute upper respiratory infections and acute sinus infections [1-7].

Microbial infections stemming from ancient times in Egypt, Greece, China, and other areas around the world are well-documented [9]. Antibiotics can and have saved millions of lives since the discovery of penicillin by Sir Alexander Fleming in 1928 [10,11]. Indeed, antibiotics have literally altered the course of modern medicine for nearly 100 years, along with saving millions of lives [9].

Unfortunately, resistance to antibiotics has also developed to nearly all antibiotics that have been developed [2]. As early as the 1950s, penicillin resistance became a clinical concern. The first case of methicillin-resistant Staphylococcus aureus (MRSA) was identified in the 1960s in both the United Kingdom and the United States [9]. The pharmaceutical industry responded by introducing many new antibiotics from the late 1960s through the 1980s, but various market forces since the 1990s have diverted pharmaceutical companies away from the development of less profitable acute care products such as new and novel antibiotics, to the more profitable chronic disease field of pharmaceutical research and development [9].

The benefits of antibiotics have been impressive. Antibiotics have saved millions of lives [2,3,5,12]. In addition to saving lives, antibiotics have been a key factor in major medical advancements in surgery, joint replacements, cardiac surgery, organ transplants, along with chronic disease management [2,3,5,12]. Antibiotics have also extended life spans by combating bacterial infections [12]. In the 1920s, the average human life span was only 56 years old; now the average US life span is nearly 80 years [12,13]. Antibiotics have had a significant positive impact throughout the world [12].

Antibiotics today, however, face serious threats due to a global antibiotic resistance crisis, largely caused by overuse and inappropriate use of these important, life-saving drugs [1]. As early as the mid-1940s, nearly 20 years after Sir Alexander Fleming’s discovery of penicillin, he himself voiced concern over the potential for antibiotic overuse when he warned that the “public will demand [the antibiotic drug] then will begin an era of abuses.” [14,15]. While bacteria have slowly developed their own resistance mechanisms and strains, epidemiological studies have demonstrated a direct relationship between antibiotic use and the emergence of bacterial resistance to available antibiotics [16].

Despite more than a decade of targeted messages from the WHO, CDC, and other reputable medical thought leaders promoting prudent antibiotic stewardship use among physicians, there hasn’t been much lasting change in prescribing habits involving antibiotics [1,2]. In fact, the CDC predicts at least 30% of all antibiotics prescribed in physician’s offices, urgent care centers, and emergency departments in the U.S. are unnecessary prescriptions [17]. Surveillance data report the emergence and dissemination of resistance among bacterial pathogens that may involve most or even all of today’s available antibiotic options [4,12], which is compounded by the lack of new antibiotics even being discovered or developed [12].

The antibiotic resistance and over-prescribing crisis, and current management measures employed to limit the use of antimicrobials and to educate healthcare prescribers, have had little to no impact in achieving an overall reduction in the use of antibiotics [4,5]. Some experts now propose that in addition to current measures involving the education of healthcare prescribers, a comprehensive education program targeted to actual end-line users, the patients themselves, will be necessary to change the public paradigm of appropriate antibiotic usage [4,5].

## Materials and methods

This Quality Improvement (QI) interventional study utilized a patient education strategy to meet its aim to evaluate the association between patient education and the patient’s satisfaction and behaviors concerning antibiotic prescriptions. The measure of antibiotic prescribing via quantitative antibiotic use metrics (number of prescriptions written) was the primary outcome, with patient satisfaction as a secondary outcome. This study used a convenience sampling of 40 hospital employees who presented for healthcare services to the hospital-based employee healthcare clinic, a primary care clinic embedded within the hospital. The S.T.E.P. process/program features straightforward, simple education via face-to-face counseling at patient encounters, along with presenting applicable printed educational pamphlets from the CDC. These two interventions were utilized in this study with research participants from the healthcare provider, with education focused on appropriate antibiotic use in the treatment of adults diagnosed with common illnesses such as an upper respiratory infection (the common cold), acute sinusitis, and acute bronchitis, which oftentimes are viral in origin. The patient-teaching process was utilized with research participants, along with a brief educational intervention delivered by the clinician as a means to curb antibiotic overuse and resistance, promote antibiotic stewardship at the consumer level, and ascertain research participant’s response to other therapeutic options rather than antimicrobial therapy as a first-line treatment, unless an antibiotic is clinically necessary as the initial therapeutic intervention, and to assess overall patient satisfaction in the encounter.

This research was conducted at an independent urgent care clinic embedded within a local acute care hospital facility where the researcher is employed. The Employee Health (EH) clinic is a free service and is only available to the hospital’s employees. It has two hospital campus locations within the local community. This study's investigator is the only healthcare prescriber at both sites, has direct patient care and patient teaching access, and is assured of consistency with data collection. Printed patient education materials provided by the CDC which focused on the appropriate use of antibiotics were utilized in this study and given to each research participant in a consistent manner, eliminating any type of bias related to data collection.

Any adult hospital employee presenting to the EH clinic with acute symptoms consistent with a common cold or upper respiratory infection (URI), acute sinusitis, and/or acute bronchitis was asked to participate in this research study during the data collection period. Direct one-on-one patient counseling by the study's clinician focused on common viral infections versus bacterial infections. These included the diagnoses of upper respiratory tract infections (URI), acute sinusitis, and acute bronchitis. Additionally, the use of printed CDC patient education materials specific to the relevant diagnoses (URI, acute sinusitis, acute bronchitis) was reviewed and given to patients in their approximate 15-minute appointment time allotment. Interested patients gave verbal informed consent for voluntary participation and authorization to participate in this study after the purpose of the study and a full description of the research was explained to each potential participant. As this research was adjunctively added to what the researcher was already doing and no additional risk or harm to the research participants was anticipated by simply offering CDC-created patient education materials to research participants, prior approval and formal presentation through an investigational review board (IRB) was not deemed to be necessary. The study’s investigator was able to accomplish the following with a study population of 40 research participants (n=40), a small but adequate sample size, following CDC antibiotic prescribing guidelines:

As a primary care provider, the researcher will continue to follow prudent, best-practice antibiotic prescribing and good stewardship of antimicrobials through prescription conservation tailored to the diagnosis, while avoiding antibiotic use for presumptive viral infections.

As a primary care provider, the researcher will engage in and provide patient education, offering technical expertise and other highly credible educational resources available through the CDC with appropriate patient encounters at the EH clinic where the researcher is employed, when research participants present with acute infections (upper respiratory infections, acute sinusitis, acute bronchitis, etc.) that by history and/or clinical findings through a focused physical examination, appear viral in nature.

The researcher will ascertain the responsiveness of the research participant to the above-referenced antibiotic educational efforts through the use of CDC’s printed patient education materials, along with the researcher’s own educational intervention during the patient encounter and with a phone call questionnaire follow-up or a return-to-clinic follow-up appointment with the same research participant/patient within a reasonable timeline, approximately two weeks after the initial encounter, to ascertain if their presenting complaint had resolved and/or what additional steps they may have taken after our initial encounter (i.e., did they end up going to a different healthcare provider who gave them an antibiotic prescription?).

The researcher provided additional patient-education materials to appropriate and applicable patients encountered in the EH clinic, which focused on healthy lifestyles that promote health and prevent infections in the first place, such as proper handwashing, along with supportive care options if they become ill with typical (usually viral) acute URI or acute sinusitis, and/or other acute symptoms. Additionally, the researcher provided information and education concerning the risks of inappropriately using antibiotics, their potential side effects such as clostridium difficile illness, antibiotic resistance, and the importance of antibiotic stewardship, as per S.T.E.P. guidelines.

The researcher maintained patient confidentiality by using the research participant’s first and last name initials, and only involved patients who verbally agreed to participate in the initial patient encounter along with a subsequent follow-up questionnaire encounter by phone call from this researcher, and/or a return-to-clinic appointment initiated by either party. Research participants' responses were kept confidential by utilizing a simple checklist format indicating what interventions were implemented with each individual research participant. The data was then analyzed after the data collection process was completed.

As the research is adjunctively added to what this author is already doing, and no additional risk or harm to the research participants is anticipated by offering CDC-created patient education materials, prior approval and formal presentation through an investigational review board (IRB) is not deemed to be necessary.

This research project’s data collection phase began after receiving approval to do so from the Research Director at Oceania University of Medicine (OUM), and continued until the goal of 40 research participants (n=40) sample size was obtained. There were no costs associated with this study.

## Results

Forty research participants agreed to participate in this intervention trial concerning antibiotic prescription rates and patient satisfaction as the two main outcome measures. All research participants gave verbal approval to patient-teaching at the time of the patient encounter in the EH clinic and to a phone call follow-up questionnaire encounter one to two weeks later. This two-part research encounter expectation was set up at the first encounter, but the research participants were, per IRB guidelines, able to decline participation in the second encounter, however, all but one research participant completed both parts of this study, achieving a 97.5% response rate. The vast majority of research participants were adult female employees (90%) at the clinical research site (Table 1).

**Table 1:** Research participant characteristics

Total # of research participants	n=40
% of Females:	36 (90%)
% of Males:	4 (10%)
Total:	40 (100%)

Most patients presented to the EH clinic with one chief complaint, but multiple complaints per patient were also recorded as some had overlapping symptoms (Table 2).

**Table 2:** Medical complaint(s) at first encounter* *More than one complaint possible, therefore the total exceeds 40 URI: upper respiratory infection

Complaints	
URI/Common cold	14
Sinus congestion/infection	11
Sore throat	8
Nasal drainage	6
Ear(s)pain/congestion	5
Nasal congestion	3
Post nasal drainage	3
Acute bronchitis	2
Fatigue	2
Headache/Sinus pressure	1

Patient satisfaction ascertained at the initial patient encounter was overwhelmingly positive. As outlined in Table 3, all but two research participants verbally stated that the patient-education they received as printed material from the CDC and the researcher’s explanation/teaching was positive and helpful. Only two antibiotics were prescribed at the initial encounter, one prescription each to two different patients. Six patients (research participants) were already on antibiotics when they presented to the EH clinic at their initial encounter.

**Table 3:** Patient satisfaction with education at initial encounter

Patient response	
Positive response to patient-teaching	38 (95%)
Negative response to patient-teaching	2 (5%)

Research participants’ overall satisfaction with their EH clinic visit was ascertained at their follow-up phone contact by the clinician-researcher one to two weeks after the initial encounter where they were asked about their overall experience (patient satisfaction, Table 3) and outcome (did they get better with the treatment plan outlined at the initial encounter or did they get worse and have an additional medical encounter?). Patient satisfaction, related to the patient-teaching they received in congruence with the S.T.E.P. guidelines for antibiotic prescribing, and the clinician-researcher’s management was assessed at the research participant’s follow-up phone contact via a verbal questionnaire format utilizing a Likert scale format for patient responses. Receiving patient-education from the researcher and whether or not an antibiotic was prescribed were both independently associated with patient satisfaction. The relationship between being given an antibiotic prescription and receiving patient education on one hand and patient satisfaction on the other hand would require multivariate logistical regression with statistical models and calculations, adjusted odds rations (ORs), and other SPSS analysis which was not done in this study. Additional variables, including patient’s prior antibiotic use and even historical expectations for receiving an antibiotic prescription are pragmatic concerns, but they require advanced statistical software and advanced analytical interpretation that are beyond the aims established for this study.

All but one research participant was involved in the second (follow-up) portion of this research study. Of the 39 patients involved at follow-up, 32 (82%) stated that they got better, defined as symptom improvement or resolution, when they were contacted via phone by the researcher within one to two weeks after their initial encounter. Of the seven (18%) remaining patients who didn’t report improvement at their follow-up encounter, only two reported worsening symptoms, which required an additional medical contact after their initial medical encounter with this clinician-researcher at the hospital’s Employee Health clinic. Both reported going to their personal primary care physician for this additional encounter and both were subsequently given an antibiotic prescription.

Research participants' responses varied at the second (follow-up) encounter. They were captured and recorded via a Likert scale for two separate assessments. Their agreement with the researcher that their illness was of a viral nature for which antibiotics aren’t used is summarized in Table 4. The research participants' overall satisfaction rating for their participation in this research study is found in Table 5.

**Table 4:** Participants' agreement that their illness is of a viral nature

1	Those who were receptive and agreeable that the illness was viral in nature	20.0%
2	Those who were partially receptive/agreeable that the illness was viral	27.5%
3	Those who were hesitant, but willing to follow the outlined treatment plan	7.5%
4	Those who were disappointed, wanting an antibiotic prescription, not received	35.0%
5	Those who were upset/mad and wanted an antibiotic prescription, not received	5.0%
6	Those for whom an antibiotic was necessary and prescribed at the initial appointment	5.0%
	Total	100%

**Table 5:** Research participants' overall satisfaction with patient-teaching encounter

Patient response	
Positive response to patient-teaching	38 (95%)
Negative response to patient-teaching	2 (5%)

## Discussion

This research study adds to medical research knowledge and to the current literature in the area of insights, attitudes, and behaviors concerning antibiotic stewardship and antibiotic resistance by applying the S.T.E.P patient-teaching principles of antibiotic stewardship. Many research participants in this study didn’t even realize that this was a topic of concern. This is troubling given that all research participants were employees at a locally owned, non-profit healthcare organization with a 640-bed total capacity, divided nearly equally between two different campuses within the same city. A hospital of this size has many resources, including a college of health sciences and a nursing school, all of which are able to provide ample teaching opportunities that may be utilized to enhance awareness of both students and employees of various healthcare-related topics, including antibiotic stewardship and antibiotic resistance.

Several research participants in this study were uncertain about what bacterial resistance was or how it related to them, both as a personal and societal problem. Participants generally felt that they played an isolated role either in the cause of the problem or in its solution. Through direct interactions with study participants, this researcher found that 35% of participants were disappointed that they didn’t receive an antibiotic, and 5% became mad/upset with this researcher, stating that they wanted/expected an antibiotic prescription when it was not received. This was even despite being given relevant and applicable printed patient education materials from the CDC, in addition to the researcher’s verbal patient-teaching interventions with an explanation as to why an antibiotic prescription was not warranted.

This research study also elicited the importance of the healthcare provider’s role in influencing and managing the necessary paradigm of change concerning antibiotic stewardship and resistance. From this study it was found that overall, 55% of research participants were receptive and agreeable to their illness being viral in nature, and the appropriate role of medications, including the use of OTC medications, was incorporated into a treatment plan rather than an antibiotic prescription. The expectations of these research participants were made explicit in the study. Healthcare consumers want to be listened to and have their needs met. They demonstrated satisfaction when given a thorough clinical examination and their findings explained to them by the healthcare provider. The majority were also receptive to this author’s treatment options, supportive cares, and suggestions for OTC medications. Generally, in all but 5% of those participating in this study, it was found that research participant’s satisfaction involving clinical decisions that were made, including the decision not to treat with antibiotics when it was not warranted, was enhanced when these issues, along with their questions answered in a respectful and complete manner. When these elements were utilized, this researcher found that 95% of study participants were open and receptive to the healthcare provider, with increased confidence and trustworthiness of the researcher. Clearly, the vast majority (95%) of study participants accepted the healthcare provider/researcher’s decision not to prescribe an antibiotic if it was clearly explained and the study participant was included in the dialogue.

This study’s results are found to be in agreement with those of other studies [18-22] where patient satisfaction with healthcare encounters was not necessarily related to the end result of an antibiotic prescription. Many just wanted the reassurance that they didn’t have a more serious illness and were satisfied with this researcher’s information presented via CDC’s printed material and with this author’s patient teaching interventions.

While randomized controlled clinical trials are widely accepted as the most reliable method of ascertaining an intervention’s effectiveness, most of these studies are able to isolate and evaluate the effects of a single, controlled intervention. This particular research study examined the design, execution, and evaluation of multiple, complex interventions, many of which are interconnected and include several factors related to the patient’s prior antibiotic use. The patients who presented to the EH clinic because of acute symptoms such as sinusitis, bronchitis, cough, sore throat, head congestion/sinusitis and other similar acute symptoms may walk in already expecting to receive a prescription for medication, such as an antibiotic. This expectation may be due to their past medical experiences, expectations, and outcomes (including obtaining an antibiotic for similar symptoms in the past). Patient teaching in the form of printed material from respected sources and clinician-lead dissemination of information and/or their reassurance is a complex and multi-factorial variable that is difficult, if not impossible, to measure directly but can help to change patient perspectives regarding appropriate antibiotic use [22]. These complex interactions and interventions present challenging difficulties in defining, developing, documenting, evaluating, statistically analyzing, and replicating research studies of this nature.

Despite these research barriers, quality improvement efforts appear to be effective at reducing both inappropriate expectations (which can be those of the patient and the prescriber) and treatment with antibiotics. This study contributes to the limited available evidence and prior research in the specific area of antimicrobial stewardship programs, but such research and programs can have an effect on a variety of outcomes at the individual and societal level, in terms of antibiotic stewardship and the slowing of antibiotic resistance [23]. Prior research has supported this and has found that no single QI strategy was more effective than others, although active clinician-presented patient education may be more effective than passive education such as giving the patient a brochure to read [24].

Whether or not the patient received patient teaching/information/reassurance was more strongly associated with positive patient satisfaction than the prescribing of an antibiotic. This study’s findings are congruent with previous research studies [18-21,23-25]. Effective quality improvement interventions can be classified as patient education, clinician education, or best-practice guidelines compliance (often with financial or regulatory incentives attached). Relevant feedback from all of these QI interventions and sources can also aid in the further expansion of knowledge and research in this specific area of antimicrobial stewardship, in addition to this study’s use of the S.T.E.P. patient education process. 

The primary outcome in this study was the number (or percentage) of patients who were appropriately prescribed an antibiotic, which occurred for two research participants, or 5.1% of the total research subjects involved. A secondary outcome included patient satisfaction as evidenced by the research participant’s verbal response obtained from a second follow-up contact. The vast majority found the applicable educational teaching for their symptoms to be helpful, with 95% of participants indicating a positive overall experience with their involvement in this study. Very few studies specific to this topic were found that address secondary outcomes [24].

The majority of participants involved in this study voiced satisfaction and reported a positive patient experience when they received information and/or reassurance during the initial encounter, regardless of an antibiotic prescription at the conclusion. Many (48.7%) were happy/satisfied just to have their own suspicions of a viral illness confirmed by a healthcare provider and were in either complete agreement (20.5%) or at least partial agreement (28.2%) that their symptoms were viral in nature, with the understanding that an antibiotic would not be either helpful or appropriate. Only one patient actually became somewhat mad and upset with this research-prescribing author that she didn’t receive an antibiotic prescription. 

This study has several limitations. It also did not sample a general population. This study utilized a convenience sample of employees at a local healthcare facility (hospital) who sought a free healthcare encounter through the hospital’s employee health clinic. This may present skewed data in at least three separate ways.

First, the clinic appointments are all free to those utilizing the services of this employee health clinic and the researcher, hence financial constraints are not barriers that are encountered at this research site as they may be in the general population.

There may also be a risk of over-utilizing this service, given its easy, wide, and free access. This researcher has no role in setting or scheduling patient appointments. The employee health clinic has had employees who have been frequent utilizers of this service. While this researcher is the only healthcare provider at this hospital and is able to prevent employees from returning to a different provider within the same EH clinic who may give them an antibiotic prescription, this may not always be the situation. Patients may “shop around” and go to different healthcare providers until they get what they want.

Another way that this study’s results may not be representative of the general population is in terms of the background and lack of diversity of its study participants, as all research participants are hospital-based employees, the majority (90%) of whom were female. Even other healthcare-based patients may hold different views than those from this specific hospital-based microcosm. Recruitment for this study was limited to the hospital population which is also not likely to be representative of the general population in terms of the education level achieved, especially given that a number of the research participants in the study were healthcare providers themselves, employed as registered nurses.

## Conclusions

Healthcare promotions and campaigns focusing on other public healthcare issues have found that simple, practical advice influences public perceptions and attitudes, and are necessary for paradigm shifts such as the important topic contained within this research on antibiotic stewardship. This study found that the healthcare prescribing provider can play a key role, using their position, knowledge, and experience, to deliver best practice, evidence-based medicine. Furthermore, through good communication skills, this research-provider was able to establish rapport, trust, and build a positive healthcare provider-patient relationship, even within a single, brief 15-minute healthcare encounter. Additionally, this study’s results demonstrate that treatment decisions can also be made within this single patient encounter that are in the best interest of the research participant, even when antibiotics are not necessary. This researcher found that 95% of study participants were satisfied with the overall healthcare encounter, and that 20% of this research population was merely seeking reassurance of their own conclusions concerning their health status and were not even expecting an antibiotic prescription.

In conclusion, scientific research, such as this study, adds to the knowledge in this area when specifically addressing the role of the clinician’s actions in terms of patient-teaching, along with the concurrent use of credible supplemental education tools offered to the patient, which helps to reinforce the message and intervention concerning antibiotic stewardship. Healthcare prescribers in primary care and acute care/urgent care settings should not bring their own possible biases forward by overestimating the influence of an antibiotic prescription on patient satisfaction, as most patients just want appropriate information and/or reassurance regarding their medical complaints. It’s also advisable to explore patients’ past experiences and expectations as to why they think that an antibiotic would be necessary for their treatment. Following the patient’s sharing of their ideas, expectations, and perspective about antibiotics, the provider can then provide appropriate and accurate information about uncomplicated acute illnesses (which tend to be viral), their self-limiting character, and use the simple S.T.E.P. teaching process to quickly explain the ineffectiveness of antibiotics with viral illnesses and the consequences of antibiotic resistance, before making shared and appropriate treatment plan decisions with the patient. This study found that all of this can be quickly and concisely covered during a typical short (15-minute) patient encounter, while also yielding high patient satisfaction results. Further research and investigation is necessary to determine the relative and lasting influence of patient-teaching interventions which focus on reducing antibiotic requests by patients and prescribing habits by healthcare providers. A larger sampling size would also be desirable, however, this research study, despite its limitations, adds to current literature in the area of antibiotic stewardship. 
